# Serum Concentrations of Polychlorinated Biphenyls in Relation to *in Vitro* Fertilization Outcomes

**DOI:** 10.1289/ehp.1002922

**Published:** 2011-02-24

**Authors:** John D. Meeker, Arnab Maity, Stacey A. Missmer, Paige L. Williams, Shruthi Mahalingaiah, Shelley Ehrlich, Katharine F. Berry, Larisa Altshul, Melissa J. Perry, Daniel W. Cramer, Russ Hauser

**Affiliations:** 1 Department of Environmental Health Sciences, University of Michigan School of Public Health, Ann Arbor, Michigan, USA; 2 Department of Statistics, North Carolina State University, Raleigh, North Carolina, USA; 3 Obstetrics, Gynecology and Reproductive Biology, Brigham and Women´s Hospital, Harvard Medical School, Boston, Massachusetts, USA; 4 Department of Epidemiology, Harvard School of Public Health, Boston, Massachusetts, USA; 5 Channing Laboratory, Department of Medicine, Brigham and Women´s Hospital, Harvard Medical School, Boston, Massachusetts, USA; 6 Department of Biostatistics, Harvard School of Public Health, Boston, Massachusetts, USA; 7 Department of Environmental Health, Harvard School of Public Health, Boston, Massachusetts, USA; 8 Vincent Memorial Obstetrics and Gynecology Service, Andrology Laboratory and in Vitro Fertilization Unit, Massachusetts General Hospital, Boston, Massachusetts, USA

**Keywords:** environment, epidemiology, female, organochlorine, reproduction

## Abstract

Background: Human exposure to polychlorinated biphenyls (PCBs) remains widespread. PCBs have been associated with adverse reproductive health outcomes including reduced fecundability and increased risk of pregnancy loss, although the human data remain largely inconclusive.

Objective: Our goal was to explore the relationship between serum PCB concentrations and early pregnancy loss among a large cohort of women undergoing *in vitro* fertilization (IVF) between 1994 and 2003.

Methods: Concentrations of 57 PCB congeners were measured in serum samples collected during 827 IVF/intracytoplasmic sperm injection cycles from 765 women. Joint statistical models that accommodate multiple outcomes and multiple cycles per woman were used to assess the relationship between serum PCB quartiles and implantation failure, chemical pregnancies (human chorionic gonadotropin level > 5.0 mIU/mL) that did not result in clinical pregnancy, or spontaneous abortion, while also adjusting for confounders.

Results: PCB-153 was the congener present in the highest concentration (median, 46.2 ng/g lipid). Increasing quartiles of PCB-153 and the sum of all measured PCB congeners (ΣPCBs) were associated with significantly elevated dose-dependent odds of failed implantation. Adjusted odds ratios (95% confidence interval) for highest versus lowest quartile were 2.0 (1.2–3.4) for PCB-153 and 1.7 (1.0–2.9) for ΣPCBs. There were suggestive trends for increased odds of implantation failure for PCB-118 and cytochrome P450–inducing congeners (*p*-values for trend = 0.06). No statistically significant associations between PCBs and chemical pregnancy or spontaneous abortion were found.

Conclusions: Serum PCB concentrations at levels similar to the U.S. general population were associated with failed implantation among women undergoing IVF. These findings may help explain previous reports of reduced fecundability among women exposed to PCBs.

Polychlorinated biphenyls (PCBs) are a class of persistent and lipophilic human-made compounds widely used in industrial and consumer products for decades before their production was banned in the United States and other developed countries in the late 1970s. PCBs remain ubiquitous environmental contaminants because of their extensive use and persistence. The half-life of PCBs in the blood ranges from < 1 to > 10 years, depending on the congener (Brown 1994; Phillips et al. 1989b). PCBs can be measured in most of the general population because of their environmental ubiquity and persistence. For example, in a recent report of a large and statistically representative sampling of 1,800 individuals ≥ 12 years of age from the U.S. population, 31 of 35 PCB congeners measured were detected in > 60% of serum samples, and 21 congeners were detected in > 95% of samples (Patterson et al. 2009). The general population is exposed primarily through ingestion of contaminated foods (e.g., fish, meat, and dairy products), although occupational, ambient, and indoor sources of exposure may exist as well (Freels et al. 2007; Harrad et al. 2006, 2009; Herrick 2010; Herrick et al. 2004, 2007; Kohler et al. 2005; Norstrom et al. 2010; Wingfors et al. 2006).

Exposure to PCBs can result in an internal dose to the female reproductive tract, as PCBs have been measured in human follicular fluid (De Felip et al. 2004; Meeker et al. 2009; Younglai et al. 2002), ovarian tissue (Mes et al. 1990), placenta, uterine muscle, and amniotic fluid (Polishuk et al. 1977), as well as in embryos and fetuses (Covaci et al. 2002; Nishimura et al. 1977), providing evidence of exposure to critical tissues during important windows of early development. PCBs have been associated with a range of adverse health effects [Agency for Toxic Substances and Disease Registry (ATSDR) 2000; Carpenter 2006], including adverse effects on reproduction (reviewed by Faroon et al. 2001; Foster 1995; Kimbrough 1995; Lione 1988; Longnecker et al. 1997; Toft et al. 2004) and increased risk of pregnancy loss (Toft et al. 2010). However, the human data available on this topic to date remain vague and largely inconclusive. In our study we explored the relationship between serum PCB concentrations and early pregnancy loss among women undergoing *in vitro* fertilization (IVF) and intracytoplasmic sperm injection (ICSI). This study population serves as a unique model to study early pregnancy end points not otherwise observable in women.

## Methods

*Study population.* Details of the main study within which the present substudy was performed have been described previously (Meeker et al. 2007a, 2007b). Briefly, between August 1994 and June 2003, couples undergoing IVF or ICSI were recruited through three Boston-area clinics to participate in a study of predictors of IVF outcomes. The main study was conducted in two phases (1994–1998 and 1999–2003) corresponding to a 5-year renewal of the study after the completion of the first 5 years. Study protocols were approved by the human research committees at Brigham and Women´s Hospital, Harvard School of Public Health, and the University of Michigan. Study subjects gave written consent before participating in the study. Approximately 65% of couples who were approached agreed to participate in the study. Most women who declined participation cited lack of time or did not want the added stress of participating. Participation did not vary greatly by clinic, and IVF outcomes did not differ by participants and nonparticipants. Couples who required donor gametes, gestational carrier, or gamete intrafallopian transfer were not eligible for enrollment. After these exclusions, 2,350 couples were enrolled in the main study. Many couples underwent multiple IVF/ICSI cycles (up to six), with the mean being two cycles per couple.

All IVF outcome variables were defined from data abstracted from the medical record by trained research nurses. Only women who proceeded to embryo transfer were eligible for the present analysis. When at least one embryo was transferred but human chorionic gonadotropin (hCG) levels did not reach 5.0 mIU/mL, the cycle outcome was defined as a failure of implantation. A chemical pregnancy was defined by a luteal hCG measurement of ≥ 5.0 mIU/mL with no further evidence (gestational sac and fetal heartbeat) of a continued pregnancy. Clinical pregnancy was determined by ultrasound visualization of a gestational sac and a fetal heartbeat. Among clinically recognized pregnancies, outcomes included in the analysis were spontaneous abortion (fetal demise before 20 weeks of gestation) and live birth of at least one infant. Numbers for the outcomes of ectopic pregnancy (gestation outside of the uterus), molar pregnancy, and stillbirth (fetal demise at 20 gestational weeks or later) lacked power to investigate independently and were excluded from further consideration in the present analysis.

Because of financial constraints, we were unable to measure PCBs in serum from all women and cycles in the study. Thus, to maximize statistical power we based our sampling strategy on the outcomes of interest (i.e., failed implantation, chemical pregnancy, and spontaneous abortion) to improve statistical power. Because failed implantation is a common occurrence, we analyzed serum PCB concentrations for a random sample of 200 women with first-cycle implantation failures. Because chemical pregnancies and spontaneous abortions were less common, all cycles from the entire cohort with these outcomes were selected. A corresponding sample of first-cycle live births was selected using stratified sampling based on age category, study center, and study phase. In addition to these selected women, a random sample of 265 repeated cycles among a subset of 110 women was selected for PCB analysis in a prior analysis of within-woman variability in serum PCB concentrations (Meeker et al. 2009), and outcome data from those cycles were also considered in the present analysis. In total, 765 women, who underwent a total 827 IVF/ICSI cycles, were included in the present analysis. The 827 cycles comprised 229 implantation failures, 177 chemical pregnancies, 124 spontaneous abortions, and 297 live births.

*Measurement of PCBs in serum.* Blood samples were collected from women at their first IVF/ICSI cycle during the follicular phase immediately before hCG administration. Our recent longitudinal analysis showed a very strong correlation between PCB concentrations measured in blood samples collected during different IVF cycles within women (Meeker et al. 2009). Thus, only a single blood sample from the first cycle of each woman was analyzed for PCBs. The serum fraction was separated for all blood samples by centrifugation for 5 min, aliquoted, and stored at –80°C until analysis. Measurement of PCBs in serum was conducted by the organic chemistry analytical laboratory, Harvard School of Public Health, using methods described previously (Hauser et al. 2003; Korrick et al. 2000). In brief, after liquid-liquid extraction and column chromatography cleanup, the samples were analyzed by gas chromatography with dual micro-electron capture detection on two capillary columns of different polarity using two internal standards. Samples were accompanied by the following quality control samples: procedural blanks, matrix spike samples, and laboratory control samples. Each sample was spiked with two surrogate compounds to monitor the efficiency of the extraction procedure. All final results were reported after subtracting the amount of the analyte measured in the procedural blank associated with the analytic batch. Results were not adjusted for surrogate recoveries. Target analytes included 57 individual PCB congeners. Method detection limits (MDLs) were determined as three times the standard deviation obtained from the analysis of the eight aliquots of pooled serum fortified with target analytes at 0.02 ng/g serum (U.S. Environmental Protection Agency 1984). The wet-weight MDL values for all PCB congeners were < 0.05 ng/g, with most of the congeners < 0.01 ng/g.

Because PCBs partition according to the lipid content of tissues, and serum lipid levels vary between fasting and nonfasting states, serum lipids also need to be considered for the valid interpretation of serum levels (Phillips et al. 1989a). Serum total cholesterol and triglycerides were measured enzymatically, and total lipids were calculated by Phillips formula (Bernert et al. 2007; Phillips et al. 1989a).

*Statistical analysis.* We explored the relationship between IVF outcomes and three individual PCB congeners (PCB congeners 118, 138, and 153), as well as the sum of all measured congeners (ΣPCBs). In addition, an analysis of the relationship between IVF outcomes and groupings of PCBs was conducted based on structural and biological activity, as previously proposed (Wolff et al. 1997). PCBs were grouped as follows: group 1, potentially estrogenic and weak phenobarbitol inducers (congeners 44, 49, 52, 101, 187, 174, 177, 157/201); group 2, potentially antiestrogenic and dioxin-like (congeners 95/66, 74, 77/110, 105/141, 118, 156, 167, 128, 138, 170); and group 3, phenobarbital, cytochrome P450 (CYP)1A, and CYP2B inducers (congeners 99, 153, 180, 196/203, 183). When forming the summed values, congeners with concentrations below the MDL were assigned a value of MDL divided by the square root of 2.0. Distributions of organochlorine concentrations in serum were tabulated and compared between the primary congeners and congener groupings of interest.

We used multivariate generalized linear regression models to assess the association of serum levels of PCB congeners and PCB groupings, categorized into quartiles, with early pregnancy failure. The data were structured to accommodate joint models for multiple outcomes and multiple cycles per woman. In each cycle, whenever a woman was at risk of having a failure due to implantation failure, chemical pregnancy, or spontaneous abortion, a binary outcome (Y) was recorded (1 = failure, 0 = not a failure). Thus, a woman could make up to three contributions to risk sets for the possible outcomes within each IVF cycle. For example, if a woman experienced implantation failure in cycle 1, and spontaneous abortion in cycle 2, but had a live birth in cycle 3, then we would consider the woman at risk only for implantation failure in the first cycle (her outcome is failure), at risk for both implantation failure and spontaneous abortion in the second cycle (she did not fail during the first possible failure point and thus continued to be at risk for the second possible failure type), and at risk for all three failure outcomes for the third cycle (but she did not fail at any of these three failure points). The correlation among both multiple failure types and among cycles for the same woman is taken into account by incorporating a random effect for each woman, which adjusts for the correlation between multiple outcomes on each woman.

We then used a logistic regression model for each early pregnancy outcome while adjusting for other covariates. To account for the possibility that the relationship between PCBs and different types of failure end point may differ, we included interaction terms between PCB quartile and failure type in the model. Similar interaction terms were also considered for age and failure type to explore the possibility of differing age effects on the various failure end points, although these interactions were not statistically significant and were removed from the final models. To account for dependence between two cycles for a particular woman, we included woman-specific random effects, which were assumed to be Gaussian. Tests for trend were conducted by assigning each PCB quartile an ordinal integer value of 0 (lowest quartile) to 3 (highest quartile).

Potential confounding variables considered in our analysis included those that may be associated with serum PCB concentrations and/or treatment outcome: serum lipids, site (of the three participating Boston-area clinics), study phase (1994–1998 or 1999–2003), race/ethnicity (Caucasian vs. other), previous live birth (yes/no), maternal age (< 35, 35–37, 38–40, > 40 years), body mass index (BMI), smoking status (never, former, or current), the number of ampules of gonadotropins delivered, ovarian stimulation protocol (downregulation vs. other), ICSI (yes/no), number of embryos transferred, and primary infertility diagnosis (tubal factor, ovulatory dysfunction, male factor, or unexplained). The use of serum levels of lipophilic compounds standardized by serum lipids (by dividing serum concentration of the compound of interest by serum lipids) as an independent variable in multivariate models has been shown to be prone to bias (Schisterman et al. 2005). Thus, we also modeled IVF outcomes in relation to wet-weight serum levels of PCBs and adjusted for serum lipids as a covariate in multivariate models, as recommended by Schisterman et al. (2005) because of the lower bias of this approach in most scenarios. Because many studies report PCB effect estimates based on lipid-standardized concentrations, we also used lipid-standardized concentrations as the exposure variable in the models for comparison in a secondary analysis.

Data analysis was conducted using SAS software (Version 9.1; SAS Institute Inc., Cary, NC). *p*-Values < 0.05 were considered statistically significant.

## Results

Data on a total of 827 IVF or ICSI cycles from 765 women were included in our analysis (Table 1). The women had a mean age of 35.9 years and were primarily Caucasian (91%) nonsmokers (92%). Almost one-quarter of the women (24%) had a previous live birth. Approximately two-thirds of the women had either unexplained infertility (34%) or were in couples diagnosed with male factor infertility (35%), and the remaining one-third of women were diagnosed with either tubal factors (19%) or ovulatory dysfunction (13%). Distributions of serum PCB concentrations are presented as both wet weight (nanograms per gram serum) and as lipid-standardized concentrations (nanograms per gram lipid) in Table 2. As expected, PCB-153 was the congener present in the highest concentration (median = 46.2 ng/g lipid; range 4.0–428 ng/g lipid) and, on average, accounted for 18% of ΣPCBs. There were strong correlations between the three primary congeners of interest, the three congener groupings, and ΣPCBs. Spearman correlation coefficients ranged from 0.62 between PCB-118 and group 1 congeners to 0.95 between PCB-153 and group 3 congeners (data not shown).

**Table 1:** Distribution of demographic, covariate, and outcome data.*a*

Table 1. Distribution of demographic, covariate, and outcome data.*a*	
Characteristic	Value
Age (years)	35.9 ± 4.21
BMI	24.2 ± 5.09
Caucasian	693 (90.6)
Current smoker	50 (6.5)
Former smoker	223 (29.2)
Previous live birth	181 (23.6)
Ampules of gonadotropins*b*	34.4 ± 18.0
Protocol (downregulation) *b,c*	604 (73.1)
ICSI procedure (yes)*b*	259 (31.3)
No. of embryos transferred*b*	3.15 ± 1.33
Lipids (mg/dL)*d*	512 ± 113
Cholesterol	162 ± 35.7
Triglycerides	93.6 ± 55.5
Infertility diagnosis	
Tubal factor	143 (18.7)
Ovulatory dysfunction	99 (12.9)
Male factor	267 (34.9)
Unexplained	256 (33.5)
IVF/ICSI outcomes*b*	
Failed implantation	229 (27.7)
Chemical pregnancy*e*	177 (21.4)
Spontaneous abortion	124 (15.0)
Other (ectopic/stillbirth)	11 (1.33)
Live birth	286 (34.6)
Values are mean ± SD or *n* (%).**a***n* = 765 women, 827 cycles. **b**Calculated from cycle-level data. **c**Ovarian stimulation protocol, coded as downregulation versus other (antagonist or flare). **d**Total lipids calculated from cholesterol and triglycerides using the Philips formula (Bernert et al. 2007; Phillips et al. 1989a). **e**Chemical pregnancy: defined as a luteal hCG measurement of ≥ 5.0 mIU/mL with no further evidence (gestational sac and fetal heartbeat) of a continued pregnancy.	

**Table 2:** Distribution of serum PCB concentrations among 765 women undergoing IVF/ICSI.

Table 2. Distribution of serum PCB concentrations among 765 women undergoing IVF/ICSI.												
PCB congener or grouping		Geometric mean		Minimum		First quartile		Median		Third quartile		Maximum
Wet weight (ng/g serum)												
PCB-118		0.083		0.014		0.052		0.080		0.123		1.34
PCB-138		0.14		0.013		0.090		0.138		0.202		1.12
PCB-153		0.23		0.015		0.16		0.24		0.34		2.33
Group 1 PCBs		0.099		0.020		0.072		0.099		0.135		0.93
Group 2 PCBs		0.44		0.098		0.296		0.434		0.619		3.62
Group 3 PCBs		0.49		0.041		0.35		0.51		0.74		5.04
∑PCB		1.32		0.32		0.95		1.32		1.81		10.3
Lipid standardized (ng/g lipid)												
PCB-118		16.6		2.80		10.5		16.0		24.7		271
PCB-138		27.3		2.71		17.7		27.5		41.5		226
PCB-153		45.4		4.03		30.4		46.2		70.1		428
Group 1 PCBs		19.7		4.07		14.2		20.0		26.9		171
Group 2 PCBs		86.8		13.1		58.0		86.3		124		766
Group 3 PCBs		97.6		8.16		65.3		101.2		150		927
∑PCB		257		41.0		182		259		367		1,887


Table 3 presents adjusted odds ratios (ORs) for failed implantation, chemical pregnancies, and spontaneous abortions in relation to PCB quartiles. Because of missing data for certain covariates, 774 cycles from 720 women contributed to the multivariate analysis (see Table 3 footnote). PCB-153 and ΣPCBs were associated with significantly elevated odds of failed implantation, and these relationships demonstrated dose-dependent trends. The second through the fourth quartiles were associated with ORs [95% confidence intervals (CIs)] of 1.1 (0.7–1.9), 1.3 (0.8–2.2), and 1.7 (1.0–2.9; *p*-value for trend = 0.03) for ΣPCBs and 1.6 (1.0–2.7), 1.6 (1.0–2.7), and 2.0 (1.2–3.4; *p*-value for trend = 0.02) for PCB-153, compared with the lowest quartile. There were suggestive trends for increased odds of implantation failure for PCB-118 (*p*-value for trend = 0.06) and group 3 congeners (*p*-value for trend = 0.06), but ORs did not follow monotonic dose–response patterns. None of the congeners or congener groupings were significantly associated with odds of chemical pregnancy or spontaneous abortion.

**Table 3:** Adjusted ORs*a* for IVF/ICSI failures in relation to serum PCB quartiles (Q) among cycles with an embryo transfer.*b*

Table 3. Adjusted ORs*a* for IVF/ICSI failures in relation to serum PCB quartiles (Q) among cycles with an embryo transfer.*b*																
		No. of cycles		No. of women		Failed implantation				Chemical pregnancy				Spontaneous abortion		
Exposure quartiles						No. of events		ORs (95% CI)		No. of events		ORs (95% CI)		No. of events		ORs (95% CI)
PCB-118																
Q1		196		185		43		Referent		54		Referent		25		Referent
Q2		196		183		59		1.81 (1.11–2.97)		38		0.72 (0.43–1.22)		34		1.50 (0.79–2.83)
Q3		190		176		71		2.34 (1.42–3.84)		38		0.89 (0.52–1.51)		33		1.70 (0.87–3.32)
Q4		192		185		61		1.60 (0.96–2.69)		47		0.91 (0.54–1.53)		41		1.29 (0.66–2.53)
*p* for trend								0.06				0.87				0.42
PCB-138																
Q1		197		184		49		Referent		45		Referent		31		Referent
Q2		198		189		59		1.42 (0.87–2.30)		41		0.98 (0.58–1.68)		25		0.68 (0.36–1.34)
Q3		185		170		62		1.51 (0.91–2.48)		46		1.18 (0.68–2.05)		30		1.10 (0.57–2.15)
Q4		194		190		64		1.39 (0.83–2.30)		45		1.10 (0.63–1.91)		38		1.14 (0.59–2.20)
*p* for trend								0.24				0.61				0.41
PCB-153																
Q1		197		188		46		Referent		45		Referent		30		Referent
Q2		193		182		57		1.63 (0.99–2.71)		42		1.04 (0.60–1.78)		26		0.89 (0.46–1.73)
Q3		192		179		59		1.59 (0.95–2.66)		46		1.09 (0.63–1.90)		33		1.18 (0.61–2.27)
Q4		192		186		72		1.99 (1.16–3.40)		44		1.17 (0.66–2.10)		35		1.13 (0.56–2.27)
*p* for trend								0.02				0.58				0.56
Group 1 PCBs																
Q1		195		186		59		Referent		41		Referent		28		Referent
Q2		194		187		50		0.87 (0.54–1.41)		46		1.06 (0.63–1.80)		34		1.20 (0.64–2.26)
Q3		195		187		57		0.99 (0.61–1.61)		44		0.97 (0.56–1.68)		30		0.83 (0.43–1.62)
Q4		190		183		68		1.19 (0.72–1.93)		46		1.13 (0.65–1.98)		32		0.93 (0.46–1.85)
*p* for trend								0.38				0.76				0.59
Group 2 PCBs																
Q1		196		185		51		Referent		46		Referent		27		Referent
Q2		197		187		56		1.27 (0.78–2.08)		41		0.91 (0.53–1.55)		27		0.91 (0.47–1.76)
Q3		191		178		60		1.44 (0.87–2.36)		41		0.97 (0.56–1.68)		39		1.66 (0.86–3.18)
Q4		190		184		67		1.38 (0.82–2.32)		49		1.17 (0.67–2.05)		31		1.07 (0.53–2.14)
*p* for trend								0.20				0.54				0.44
Group 3 PCBs																
Q1		198		189		46		Referent		45		Referent		30		Referent
Q2		191		180		57		1.44 (0.88–2.38)		39		0.90 (0.53–1.55)		30		1.01 (0.53–1.93)
Q3		193		178		59		1.42 (0.85–2.38)		51		1.26 (0.74–2.17)		27		0.94 (0.48–1.85)
Q4		192		187		71		1.75 (1.03–2.97)		42		1.02 (0.57–1.84)		37		1.13 (0.57–2.25)
*p* for trend								0.05				0.65				0.79
∑PCB																
Q1		197		187		46		Referent		42		Referent		28		Referent
Q2		194		186		52		1.13 (0.68–1.85)		43		1.08 (0.69–1.85)		33		1.19 (0.63–2.26)
Q3		193		182		58		1.33 (0.81–2.18)		48		1.34 (0.78–2.31)		32		1.36 (0.70–2.65)
Q4		190		184		73		1.70 (1.02–2.85)		44		1.27 (0.71–2.29)		31		1.07 (0.53–2.15)
*p* for trend								0.03				0.30				0.75
**a**Joint models for multiple outcomes and multiple cycles per woman were used. Models were adjusted for serum lipids, study site, study phase, race/ethnicity, previous live birth, maternal age, BMI, smoking status, ampules of gonadotropins, protocol, ICSI, number of embryos transferred, and primary infertility diagnosis. **b**There were 827 cycles with serum samples analyzed for PCB concentrations. However, because of missing covariates, only 774 cycles from 720 women contributed to the multivariate analysis. Missing ethnicity, infertility diagnosis, and previous live birth were recoded as a separate category so that cycles missing this information were in the multivariate models. If a cycle was missing any other covariate, that cycle was dropped from the multivariate analysis. Specifically, 13 cycles were missing lipids, 2 cycles were missing ampules of gonadotropins, 32 cycles were missing number of embryos transferred, and 6 cycles were missing BMI.																

As a potentially more clinically relevant measure of effect, we also calculated the odds of a live birth in relation to serum PCB quartiles (Table 4). Quartiles of PCB-153 again followed a dose-related trend, where the odds of live birth were reduced by 12%, 35%, and 41% in quartiles 2–4, respectively, compared with the lowest PCB-153 quartile (*p*-value for trend = 0.03). The third and fourth ΣPCB quartiles were also associated with 41% reductions in the odds of a live birth (*p*-value for trend = 0.02). Reductions in the odds of live birth were observed for PCB-118 and for group 2 and group 3 congeners, but these relationships were not statistically significant.

**Table 4:** Adjusted ORs (95% CIs)*a* for live birth in relation to serum PCB quartiles (Q) among cycles with an embryo transfer.*b*

Table 4. Adjusted ORs (95% CIs)*a* for live birth in relation to serum PCB quartiles (Q) among cycles with an embryo transfer.*b*														
Exposure quartiles		PCB-118		PCB-138		PCB-153		Group 1 PCBs		Group 2 PCBs		Group 3 PCBs		∑PCB
Q1		Referent		Referent		Referent		Referent		Referent		Referent		Referent
Q2		0.73 (0.48–1.11)		0.95 (0.62–1.45)		0.88 (0.56–1.37)		0.91 (0.59–1.42)		0.87 (0.57–1.35)		0.89 (0.57–1.39)		0.75 (0.49–1.16)
Q3		0.51 (0.32–0.81)		0.68 (0.42–1.07)		0.65 (0.41–1.05)		0.97 (0.61–1.52)		0.59 (0.37–0.94)		0.69 (0.43–1.12)		0.59 (0.37–0.94)
Q4		0.74 (0.47–1.15)		0.73 (0.45–1.16)		0.59 (0.35–1.00)		0.79 (0.49–1.26)		0.68 (0.42–1.11)		0.67 (0.44–1.13)		0.59 (0.35–0.98)
*p* for trend		0.09		0.57		0.03		0.40		0.05		0.08		0.02
**a**Joint models for multiple outcomes and multiple cycles per woman were used. Models were adjusted for serum lipids, study site, study phase, race/ethnicity, previous live birth, maternal age, BMI, smoking status, ampules of gonadotropins, protocol, ICSI, number of embryos transferred, and primary infertility diagnosis. **b***n *= 720 women, 744 cycles.														

Because many studies standardize PCB concentrations for lipid content before analyzing the data, we conducted a secondary analysis using lipid-standardized values in the models. Overall results were similar to those presented [see Supplemental Material, Table 1 (doi:10.1289/ehp.1002922)]. The primary differences were that the associations for PCB-118 and group 3 congeners with failed implantation and live birth became slightly stronger, whereas the relationships between ΣPCBs and these outcomes were slightly weakened.

## Discussion

In our study we report that serum concentrations of PCBs in women may be associated with adverse IVF/ICSI outcomes. In particular, we found that the odds of failed implantation were doubled, and the odds of a live birth were reduced by 41%, among women in the highest serum PCB-153 quartile compared with women in the lowest PCB-153 quartile. Serum ΣPCBs, which was strongly correlated with PCB-153, was also associated with implantation failure and reduced odds of a live birth.

As far as we are aware, this is the first human study to assess the relationship between PCB exposure and failed implantation, although two small studies have assessed the relationship between PCBs in follicular fluid and other IVF outcomes (e.g., fertilization rate) (Jirsova et al. 2010; Younglai et al. 2002). Because implantation failure is common and occurs before a pregnancy is recognized among couples conceiving spontaneously (Chard 1991; Norwitz et al. 2001), our findings may be comparable with those in epidemiologic studies of PCB exposure and time to pregnancy (TTP). However, TTP as an outcome lacks specificity, because it may represent one or more aspects of female or male factors. Several studies have reported an association between PCB exposure and increased TTP. A study of 186 women exposed to high concentrations of PCBs in the 1978–1979 Taiwanese cooking oil contamination incident reported a fecundability ratio of 0.90 (95% CI, 0.80–1.00) compared with a group of 226 reference women (Yang et al. 2008). The Collaborative Perinatal Project, which included 390 U.S. women who were pregnant between 1959 and 1965, also reported a reduced fecundability odds ratio (FOR) of 0.65 (95% CI, 0.36–1.18) among women in the highest serum ΣPCB category (> 5 µg/L) compared with women in the lowest exposure category (Law et al. 2005). In the first human study to assess preconception serum PCB concentrations and TTP, Buck Louis et al. (2009) reported FORs of 0.32 (95% CI, 0.03–3.89) and 0.01 (< 0.00–1.99) for estrogenic and antiestrogenic PCB congener groupings, respectively, among a subset of 81 women planning pregnancies who were followed for a total of 444 menstrual cycles in the New York State Angler Cohort Study. A number of earlier studies examined organochlorine exposure through consumption of contaminated fish in relation to TTP, with conflicting results (Arakawa et al. 2006; Axmon et al. 2000b, 2001, 2002, 2004, 2006; Buck et al. 2000). Limitations in many of these studies include the use of exposure surrogates instead of exposure biomarkers, self-recall of TTP, and the inclusion of only women who achieved clinical pregnancy or whose pregnancies resulted in a live birth. Our observation of a relationship between PCBs and failed implantation is consistent with the more recent and rigorously designed TTP studies showing an inverse relationship between PCBs and fecundability. Although the women in those studies had serum PCB concentrations that were much higher than the women in our study, our findings add to those studies by providing insight into a specific aspect and time point within early postconception that contributes to TTP and may be particularly sensitive to PCB exposure.

In this study, the lack of association between PCBs and risk of spontaneous abortion was consistent with a recent study of 1,344 pregnancies in Michigan women that reported no association between serum PCB concentrations > 5 ng/g wet weight and risk of miscarriage (Small et al. 2007) but was inconsistent with a recent European study of 1,710 women that reported an association between PCB-153 at serum concentrations of > 200 ng/g lipid and increased risk of fetal loss (miscarriages or stillbirths) (Toft et al. 2010). Several earlier studies also reported evidence for a relationship between PCBs and miscarriage (Bercovici et al. 1983; Leoni et al. 1989; Tsukimori et al. 2008), whereas other earlier studies reported no associations (Axmon et al. 2000a; Dar et al. 1992; Khanjani and Sim 2007; Mendola et al. 1995; Sugiura-Ogasawara et al. 2003). Many of the early studies were limited by small sample sizes or indirect exposure assessments (e.g., consumption of contaminated fish). In addition, study designs and PCB exposure levels between studies have differed greatly. Our data do not support the presence of an association between PCBs at levels similar to those among the general population and fetal loss. However, the overall body of evidence remains inconsistent, and the generalizability of our findings to highly exposed populations or women not undergoing assisted reproduction are unknown, so this relationship may require further inquiry.

Our observation of an association between PCB exposure and implantation failure is consistent with studies from the experimental literature, many of which were conducted with low and environmentally relevant dose levels (ATSDR 2000). Animal studies have demonstrated that PCBs cause reduced oocyte maturation (reviewed by Pocar et al. 2006), increased embryo degeneration and decreased embryo cell proliferation, blastocyst formation and blastocyst development, and decreased IVF success rates (Campagna et al. 2001, 2002; Kholkute and Dukelow 1997; Kholkute et al. 1994a, 1994b; Krogenaes et al. 1998; Kuchenhoff et al. 1999; Lindenau and Fischer 1996; Pocar et al. 2001), along with increased implantation failures (Linder et al. 1974), increased resorptions (Arnold et al. 1995), and decreased litter production (Jonsson et al. 1975; Seiler et al. 1994). PCB impacts on uterine receptivity may also contribute to an increased risk of implantation failure. For example, consistent with animal research (ATSDR 2000), some epidemiologic studies have reported associations between PCB exposure and altered menstrual cycles (Chao et al. 2007; Cooper et al. 2005; Mendola et al. 1997; Toft et al. 2004, 2008; Yu et al. 2000) and endometriosis (Anger and Foster 2008; Gerhard et al. 1999; Heilier et al. 2008; Porpora et al. 2006, 2009).

Our study has a number of strengths. First, it was a prospective study with exposure biomarkers and outcome measures collected at or near the likely time window of interest, which represents an improvement over many of the previous studies of PCBs and fertility or pregnancy outcomes that relied on retrospective study designs. For example, some previous studies have used exposure surrogate measures, such as self-reported contaminated fish intake, which may introduce more exposure measurement error than the use of exposure biomarkers. This study used detailed, clinically assessed data on outcome measures, whereas many previous studies relied on participant recall and may have been susceptible to recall bias. Second, we were able to assess early end points that are impossible or difficult to observe in conventional epidemiologic studies. Third, this study was large and used outcome data from repeated IVF cycles, which resulted in good statistical power to detect associations between serum PCB concentrations and failed implantation, chemical pregnancies, and spontaneous abortions. Finally, most previous studies have been conducted among populations with elevated PCB exposures, whereas women in our study had serum PCB concentrations that are more representative of the general population. For example, median (75th percentile) PCB-153 concentrations in serum samples collected between 1994 and 2003 in this study were 46 (70) ng/g lipid compared with 35 (63) ng/g lipid and 24 (47) ng/g lipid among adults ≥ 20 years of age from the National Health and Nutrition Examination Survey (NHANES) 2001–2002 and NHANES 2003–2004, respectively (Centers for Disease Control and Prevention 2009).

Our study also has a number of limitations. First, as with nearly all environmental epidemiologic studies, there may be unmeasured confounding or coexposures that may explain the observed associations. For example, if fish consumption is the primary source of PCB-153, it is possible that other exposures or activities related to fish consumption are confounding the relationship of PCB-153 with failed implantation. Second, because our study was conducted among women undergoing IVF, the extent to which we can generalize our findings to wider populations is not clear. For our results to lack generalizability, women undergoing IVF would have to differentially respond to PCB exposure compared with women conceiving spontaneously. There is currently no evidence suggesting these women may be more susceptible to PCB exposure, although it is possible that PCB exposure may contribute to a couple’s fecundability and need for IVF treatment. However, even if women undergoing IVF represent a subgroup particularly sensitive to PCBs, our findings could have important implications, because couples seeking fertility treatment represent a large and growing proportion of the population. Nevertheless, the consistency between our findings and human TTP studies suggests that our results may be generalizable. In addition, because our results are consistent with animal studies, they may provide further evidence that couples undergoing IVF provide a unique opportunity to study detailed reproductive end points not normally observable in humans. Another limitation of the present analysis is that we did not include earlier measures that are observable in couples undergoing IVF, such as oocyte quality, failed fertilization, and embryo quality. Because animal studies suggest that these end points may also be adversely affected by PCBs, these outcomes should be assessed in relation to serum PCB concentrations in the future. Studies that assess more detailed outcome measures may also provide additional clues for the potential mechanisms of action involved in the relationships reported here, which currently remain unclear. Finally, we did not have exposure data on male partners. There have been several reports of an association between PCB exposure and reduced semen quality (Meeker and Hauser 2010), which may also contribute to increased TTP and poor embryo quality in relation to PCB exposure.

In conclusion, we found that serum PCB concentrations representative of those measured among the U.S. general population were associated with increased odds of failed implantation among women undergoing IVF. These findings may help explain previous reports of reduced fecundability and increased TTP among women exposed to PCBs.

## Supplemental Material

(88 KB) PDF
