# Supplementary material for: Serum Concentrations of Polychlorinated Biphenyls in Relation to in Vitro Fertilization Outcomes
Source: Environ Health Perspect. 2011 Feb 24;119(7):1010–6. doi: 10.1289/ehp.1002922 (PMC3222973; doi:10.1289/ehp.1002922)
Supplement: (88 KB) PDF [file ehp.1002922.s001.pdf]

Supplemental Material

Serum Concentrations of Polychlorinated Biphenyls (PCBs) in Relation to *in Vitro* Fertilization (IVF) Outcomes

John D. Meeker[1], Arnab Maity[2], Stacey A. Missmer[3,4,5], Paige L. Williams[6], Shruthi Mahalingaiah[3], Shelley Ehrlich[7], Katharine F. Berry[3], Larisa Altshul[7], Melissa J. Perry[7], Daniel W. Cramer[3], Russ Hauser[7,8]

[1]Department of Environmental Health Sciences, University of Michigan School of Public Health, Ann Arbor, MI
[2]Department of Statistics, North Carolina State University, Raleigh, NC
[3]Obstetrics, Gynecology and Reproductive Biology, Brigham and Women's Hospital, Harvard Medical School, Boston, MA
[4]Department of Epidemiology, Harvard School of Public Health, Boston, MA
[5]Channing Laboratory, Department of Medicine, Brigham and Women's Hospital, Harvard Medical School, Boston, MA
[6]Department of Biostatistics, Harvard School of Public Health, Boston, MA
[7]Department of Environmental Health, Harvard School of Public Health, Boston, MA
[8]Vincent Memorial Obstetrics and Gynecology Service, Andrology Laboratory and In Vitro Fertilization Unit, Massachusetts General Hospital, Boston, MA

Address correspondence to:

John Meeker, ScD
Department of Environmental Health Sciences
University of Michigan School of Public Health
6635 SPH Tower
109 S. Observatory St.
Ann Arbor, MI 48109
Phone: 1-734-764-7184
Fax: 1-734-936-7283
Email: meekerj@umich.edu

Supplemental Material, Table 1. Adjusted odds ratios (95% confidence intervals)[a] for IVF/ICSI failures in relation to lipid-standardized serum PCB quartiles among cycles with an embryo transfer[b].

Exposure Quartiles	Failed Implantation	Chemical Pregnancy	Spontaneous Abortion
PCB 118			
Q1	Referent	Referent	Referent
Q2	1.43 (0.88, 2.32)	0.91 (0.55, 1.52)	2.03 (1.09, 3.79)
Q3	1.83 (1.11, 3.00)	0.89 (0.53, 1.51)	2.00 (1.03, 3.87)
Q4	1.42 (0.86, 2.33)	1.06 (0.64, 1.76)	1.57 (0.81, 3.08)
p for trend	0.04	0.86	0.17
PCB 138			
Q1	Referent	Referent	Referent
Q2	1.12 (0.69, 1.81)	0.93 (0.56, 1.56)	1.04 (0.55, 1.96)
Q3	1.45 (0.87, 2.40)	1.11 (0.66, 1.88)	1.62 (0.85, 3.05)
Q4	1.25 (0.75, 2.07)	0.96 (0.56, 1.65)	1.25 (0.65, 2.39)
p for trend	0.13	0.96	0.30
PCB 153			
Q1	Referent	Referent	Referent
Q2	1.69 (1.02, 2.79)	1.14 (0.68, 1.92)	0.77 (0.41, 1.45)
Q3	1.52 (0.89, 2.60)	1.08 (0.63, 1.84)	0.98 (0.52, 1.84)
Q4	2.03 (1.19, 3.46)	0.99 (0.56, 1.75)	1.01 (0.52, 1.95)
p for trend	0.005	0.98	0.80
Group 1PCBs			
Q1	Referent	Referent	Referent
Q2	1.06 (0.67, 1.69)	1.16 (0.68, 1.95)	1.26 (0.70, 2.30)
Q3	1.17 (0.72, 1.89)	1.17 (0.69, 1.99)	1.32 (0.70, 2.40)
Q4	1.28 (0.79, 1.07)	1.15 (0.66, 2.01)	0.84 (0.43, 1.63)
p for trend	0.15	0.60	0.75
Group 2 PCBs			
Q1	Referent	Referent	Referent
Q2	1.07 (0.65, 1.74)	0.86 (0.51, 1.45)	1.36 (0.73, 2.53)
Q3	1.42 (0.85, 2.35)	1.22 (0.72, 2.08)	1.52 (0.78, 2.95)
Q4	1.27 (0.76, 1.10)	0.90 (0.52, 1.57)	1.23 (0.63, 2.40)
p for trend	0.11	0.93	0.50

Supplemental Material, Table 1 (cont.)

Exposure Quartiles	Failed Implantation	Chemical Pregnancy	Spontaneous Abortion
Group 3 PCBs			
Q1	Referent	Referent	Referent
Q2	1.40 (0.85, 2.28)	1.24 (0.73, 2.11)	0.90 (0.48, 1.68)
Q3	1.40 (0.83, 2.37)	1.73 (1.01, 2.97)	1.02 (0.53, 1.98)
Q4	1.61 (0.96, 2.71)	0.92 (0.51, 1.66)	1.05 (0.56, 1.99)
p for trend	0.04	0.83	0.78
∑PCB			
Q1	Referent	Referent	Referent
Q2	1.49 (0.90, 2.47)	0.94 (0.55, 1.60)	0.79 (0.42, 1.48)
Q3	1.79 (1.08, 2.98)	1.35 (0.79, 2.30)	1.25 (0.65, 2.40)
Q4	1.51 (0.89, 2.55)	0.85 (0.49, 1.50)	0.93 (0.49, 1.78)
p for trend	0.04	0.93	0.84

[a]Joint models for multiple outcomes and multiple cycles per woman were used. Models adjusted for study site, study phase, race/ethnicity, previous livebirth, maternal age, BMI, smoking status, ampules of gonadotropins, protocol, ICSI, number of embryos transferred, and primary infertility diagnosis.
[b]There were 827 cycles with serum samples analyzed for PCB concentrations. However, due to missing covariates, only 774 cycles from 720 women contributed to the multivariate analysis. Missing ethnicity, infertility diagnosis, and previous live birth were recoded as a separate category so that cycles that were missing this information were in the multivariate models. If a cycle was missing any other covariate, that cycle was dropped from the multivariate analysis. Specifically, 13 cycles were missing lipids, 2 cycles were missing amps of gonadatropins, 32 cycles were missing number of embryos transferred, and 6 cycles were missing BMI
